# Scalable bioreactor manufacturing of iPSC-derived megakaryocytes with Proanthocyanidin A supplementation: a strategic platform for military blood supply resilience

**DOI:** 10.1016/j.mmr.2026.100057

**Published:** 2026-08-03

**Authors:** Rabea Dettmer, Alice Rovai, Willem Wolkers, Rainer Blasczyk, Constanca Figueiredo

**Affiliations:** Institute of Transfusion Medicine and Transplant Engineering, Hannover Medical School, 30625 Hannover, Germany; Biostabilization laboratory - Lower Saxony Centre for Biomedical Engineering, Implant Research and Development, University of Veterinary Medicine Hannover, 30559 Hannover, Germany; Unit for Reproductive Medicine - Clinic for Horses, University of Veterinary Medicine Hannover, 30559 Hannover, Germany; Institute of Transfusion Medicine and Transplant Engineering, Hannover Medical School, 30625 Hannover, Germany

**Keywords:** Military transfusion medicine, iPSC-derived megakaryocytes, Stirred bioreactor, Platelet manufacturing, Proanthocyanidin A (ProA), Blood supply resilience

Dear Editor,

Massive hemorrhage remains the leading preventable cause of death in both civilian trauma and combat casualties [1]. In prolonged field care and deployment scenarios, platelet (PLT) availability is particularly vulnerable due to their short shelf life and dependence on continuous donor supply chains [2]. Strengthening transfusion resilience for military operations therefore requires scalable, donor-independent manufacturing strategies compatible with decentralized or strategically buffered production. Here, we describe the implementation of a stirred-suspension bioreactor platform for large-scale generation of induced pluripotent stem cell (iPSC)-derived megakaryocytes (MKs), integrating cost-reduction measures to enhance strategic feasibility for defense medical logistics.

ProA is a naturally occurring polyphenolic compound found in several plant sources, including peanut skins and cranberries [3]. Previous studies have suggested that polyphenols, including proanthocyanidins such as ProA, can promote MK differentiation and PLT production through activation of signaling pathways involved in megakaryopoiesis, including Janus kinase (JAK)/signal transducer and activator of transcription (STAT), mitogen-activated protein kinase (MAPK)/extracellular signal-regulated kinase (ERK), and phosphatidylinositide 3-kinases (PI3K)/protein kinase B (Akt) signaling [4], [5]. These pathways are known to contribute to TPO-mediated MK proliferation, maturation, and PLT formation. Emerging evidence suggests that megakaryopoiesis and PLT production can be regulated by signaling pathways beyond the canonical TPO/myeloproliferative leukemia protein (the TPO receptor) axis [6]. Accordingly, the ability of ProA to support megakaryocytic differentiation under reduced TPO conditions may involve alternative regulatory mechanisms, although the precise molecular pathways remain to be elucidated.

The iPSC line used in this study was generated in-house from a healthy female donor with blood group O Rh-negative using Sendai virus-based reprogramming (CytoTune™-iPS 2.0 Sendai Reprogramming Kit, Thermo Fisher Scientific, USA). Prior to differentiation, the iPSC line was validated for pluripotency, trilineage differentiation potential, and normal karyotype. Differentiation of MKs from iPSCs was performed according to a previously established protocol (**Additional file 1: Methods**). The process was conducted in the DASbox® Mini-Bioreactor System (Fig. 1**a**). To reduce recombinant TPO, ProA (MedChemExpress, USA) was implemented as a potential cost-reduction strategy. ProA was dissolved in DMSO prior to supplementation of the differentiation medium. At the applied working concentration (1 µmol/L), the final DMSO concentration remained below levels reported to affect stem cell differentiation [7], [8], therefore, no separate vehicle control was included. Preliminary optimization experiments (**Additional file 1:** Fig. S1) demonstrated that reducing thrombopoietin (TPO) supplementation to 0.5×TPO (25 ng/ml) in combination with 1 µmol/L ProA maintained MK frequency and MK yield comparable to 1×TPO while showing a trend toward improved cell viability. Based on these findings, differentiation was performed under modified culture conditions consisting of reduced TPO supplementation (0.5×TPO, 25 ng/ml) and the addition of 1 µmol/L ProA (Fig. 1**b**), which were subsequently used throughout the study.

**Fig. 1:**
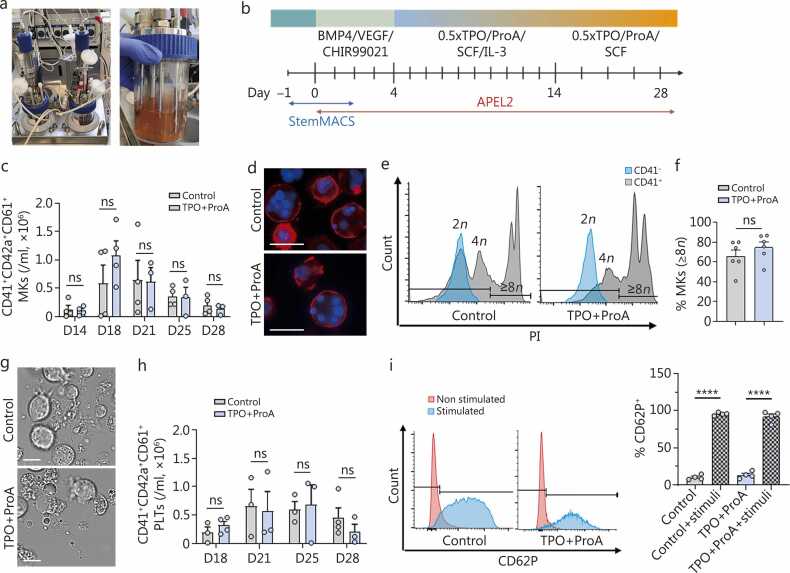
Optimized large-scale production of iPSC-derived functional MKs. **a** Photographs of the DASbox® Mini Bioreactor system used for large-scale MK production. **b** Representative schematic of the optimized MK differentiation protocol. **c** Quantification of CD41⁺CD42a⁺CD61⁺ MKs/ml of differentiation culture over 28 d, comparing the standard protocol to the modified protocol with reduced 0.5×TPO and 1 µmol/L ProA supplementation. *n*=3–4. **d** Immunofluorescence analysis showing polyploidy of bioreactor-derived MKs with CD41 (red) and DAPI (blue) for control and TPO+ProA conditions. Scale bar=20 µm. **e** Representative analysis and gating strategy of the polyploidy staining with propidium iodide for control and TPO+ProA conditions. CD41^−^ cells were used as an internal diploid (2 *n*) DNA content reference population to calibrate the DNA content analysis. This reference was used to define the baseline 2 *n* peak. CD41^+^ cells represent the MK population, which undergoes endomitosis and therefore exhibits increased DNA content, resulting in distinct ploidy peaks corresponding to 2 *n*, 4 *n*, and ≥8 *n*. **f** Comparative flow cytometric analysis of polyploid MKs ≥8 *n* in control conditions and with reduced 0.5×TPO and 1 µmol/L ProA supplementation. *n*=5. **g** Representative microscopic images of ProPLT-forming MKs for control and TPO+ProA conditions. Scale bars=10 µm. **h** Quantification of CD41⁺CD42a⁺CD61⁺ PLTs/ml of differentiation culture over 28 d, comparing the standard protocol to the modified protocol with reduced 0.5×TPO and 1 µmol/L ProA supplementation. *n*=3–4. **i** Representative flow cytometric analysis of PLT activation marker CD62P before and after stimulation with ADP and thrombin for control and TPO+ProA conditions. Comparative flow cytometric analysis of the PLT activation marker CD62P after stimulation of PLTs with ADP and thrombin harvested from control conditions and with reduced TPO and ProA supplementation. *n*=4. Statistical significance was assessed using multiple unpaired two-tailed *t*-tests (**c, h**), unpaired *t*-tests (**f**), and the ordinary one-way ANOVA followed by Tukey’s multiple comparisons test (**i**). Data represent mean±standard error of mean (SEM). ⁎⁎⁎⁎*P*<0.0001, ns non-significant. iPSC. Induced pluripotent stem cell; MK. Megakaryocyte; TPO. Thrombopoietin; ProA. Proanthocyanidin A; PLT. Platelet; ProPLT: Proplatelet; BMP4. Bone morphogenetic protein 4; VEGF. Vascular endothelial growth factor; SCF. Stem cell factor; APEL2. APEL2 medium; IL-3. Interleukin-3.

MKs generated under both standard and ProA-supported conditions exhibited a predominantly CD41⁺CD42a⁺CD61⁺ phenotype, confirming their megakaryocytic identity. The cumulative MK yield obtained from one differentiation run (harvests on D14, D18, D21, D25, and D28) was (1.90±1.57)×10⁶ MKs/ml of culture volume under standard conditions. Under conditions with 0.5×TPO reduction and 1 µmol/L ProA supplementation, the cumulative yield increased to (2.53±1.44)×10⁶ MKs/ml of culture volume, indicating preserved and numerically increased output (Fig. 1**c**). Furthermore, expression of CD42a, a marker of mature MKs and PLTs, was comparable between conditions (Fig. 1**c**), indicating preserved megakaryocytic maturation.

For military application, reduction of high-cost recombinant cytokines is not merely economical but strategically relevant. In large-scale manufacturing scenarios, whether centralized in national defense facilities or integrated into strategic medical stockpiling, cytokine expenses significantly influence scalability [9]. The successful incorporation of ProA suggests that small-molecule pathway modulators can support robust megakaryopoiesis while reducing dependence on expensive biologics.

A critical determinant of PLT production capacity is MK polyploidization and proplatelet (ProPLT) formation [10]. Polyploidy was evaluated at D18 and D21 of differentiation, as these time points consistently showed the highest MK yields during the differentiation process and therefore represent the most relevant potential harvest time points for the iPSC-derived MKs intended for transfusion. Samples from both time points were combined for analysis and are presented as a pooled dataset. No significant differences in MK polyploidy were observed between control and ProA conditions, as determined by flow cytometric analysis of DNA content using propidium iodide staining (Fig. 1**d-f**). The proportion of highly polyploid MKs (≥8 *n*) was comparable between standard differentiation conditions and reduced TPO conditions supplemented with ProA (66.12%±15.14% vs. 75.02%±13.18%, respectively) (Fig. 1**f**). To confirm terminal MK maturation, we assessed ProPLT formation, a hallmark of mature MKs. ProPLT formation was observed under both conditions (Fig. 1**g**), however, no formal quantitative analysis of ProPLT formation was performed. Instead, cumulative PLT release into the culture medium was quantified over time as a functional readout of thrombopoietic activity. Similar to MK yield calculation, PLTs collected from individual harvests were summed and normalized to the culture volume. This analysis revealed comparable thrombopoietic activity between standard and ProA-supported cultures [(1.89±1.29)×10⁶ PLTs/ml vs. (1.78±1.57)×10⁶ PLTs/ml culture volume, respectively] (Fig. 1**h**).

Given that PLT counts alone do not provide information on the functional integrity of the generated PLTs, we next examined their responsiveness to activation stimuli. PLTs collected from culture supernatants on D18 and D21, corresponding to peak MK production, were separated from larger MKs by sequential centrifugation and quantified by flow cytometry using CD41, CD42a, and CD61. Upon stimulation with thrombin and ADP, CD62P expression increased under both culture conditions, reaching 95.65%±2.15% in PLTs generated under standard differentiation conditions and 91.95%±7.66% in PLTs generated under ProA-supported conditions, demonstrating that PLTs from both conditions remained functionally responsive to agonist stimulation (Fig. 1**i**).

Overall, these findings indicate that iPSC-derived PLTs are capable of robust functional responses. While the present study demonstrates the feasibility of generating mature and functional MKs under ProA-supported differentiation conditions, the molecular mechanisms underlying the effects of ProA during iPSC-derived megakaryopoiesis remain incompletely understood. Importantly, the primary objective of this study was the generation of mature MKs for potential transfusion applications rather than the optimization of *in vitro* PLT production. Therefore, no specific measures were taken to stimulate or maximize PLT release from MKs during culture. As transfused MKs are expected to complete PLT production *in vivo* after administration, the number of PLTs released into the culture medium likely underestimates the overall thrombopoietic potential of the generated MKs. Consequently, the actual PLT yield per MK may differ from the PLT numbers detected *in vitro.* In addition, the thrombopoietic potential and functionality of the generated MKs have not yet been evaluated *in vivo* and will require further investigation in future studies. From a defense medical perspective, the scalability of this system is particularly significant. Given that a single iPSC-derived MK could release approximately 100–1000 PLTs *in vivo*, manufacturing volumes between 100 ml and 1 L are expected to be sufficient to produce transfusion-relevant PLT doses. Closed, monitored stirred bioreactors provide a controlled production environment compatible with Good Manufacturing Practice principles and strategic reserve manufacturing.

In military contexts characterized by disrupted supply chains, extended evacuation times, or multinational deployment environments, autologous or universal iPSC-derived PLT production platforms may complement or even overcome conventional donor-based logistics. While further scale-up and regulatory validation are required, the present data demonstrate technical feasibility of high-yield, maturation-competent MK production under scalable, economically optimized conditions.

In summary, we established a controlled stirred-suspension bioreactor platform capable of producing polyploid, functional iPSC-derived MKs at clinically relevant scale. Partial reduction of TPO and supplementation with ProA preserves yield and maturation while improving economic feasibility. This integrated approach represents a promising strategic component for enhancing resilience of military transfusion medicine and future donor-independent PLT supply systems.

## Abbreviations

DO: Dissolved oxygen
iPSC: Induced pluripotent stem cell
MK: Megakaryocyte
PLT: Platelet
ProA: Proanthocyanidin A
ProPLT: Proplatelet
SCF: Stem cell factor
TPO: Thrombopoietin

## Declarations

None

## Ethics approval and consent to participate

The study was approved by the Ethics Committee of the Medizinische Hochschule Hannover. Written informed consent was obtained from all donors prior to the derivation of induced pluripotent stem cells.

## Authors’ contributions

RD and CF contributed to the study concept and design, methodology, data analysis, investigation, validation, and drafting of the manuscript. AR contributed to the study concept and design, methodology, and data analysis. CF and RB were responsible for funding acquisition. WW and RB provided scientific advice and contributed to the review and editing of the manuscript. All authors read and approved the final manuscript.

## Funding

This work was supported by the Federal Office of Bundeswehr Equipment, Information Technology and Use (BAAINBw MHH-HF-ZB3-2024).

## Declaration of Competing Interest

The authors declare that they have no competing interests.

## Data Availability

The datasets used and/or analysed during the current study are available from the corresponding author on reasonable request.

## Footnotes

Appendix A Supplementary data associated with this article can be found in the online version at doi:10.1016/j.mmr.2026.100057.
