# Supplementary material for: Scalable bioreactor manufacturing of iPSC-derived megakaryocytes with Proanthocyanidin A supplementation: a strategic platform for military blood supply resilience
Source: Mil Med Res. 2026 Aug 3;13(1):100057. doi: 10.1016/j.mmr.2026.100057 (PMC13470163; doi:10.1016/j.mmr.2026.100057)
Supplement: Supplementary file 1 [file mmc1.pdf]

Methods

Culture conditions and differentiation protocol

The differentiation of MKs from iPSCs was carried out based on a previously published protocol [1]. Critical process parameters, including dissolved oxygen (DO), pH, and temperature (37 ℃), were continuously monitored and regulated to ensure reproducibility and process control. Cultures were aerated with 21% O_2 and 5% CO_2, the pH was maintained at 7.0. Additional oxygen was supplied only when required to maintain DO above 30%. Briefly, iPSCs were seeded in StemMACS™ iPS-Brew XF medium (Miltenyi Biotec, Germany) supplemented with 10 μmol/L Y-27632 (Stemcell Technologies, Canada). Differentiation was initiated using STEMdiff™ APEL™2 medium (Stemcell Technologies), containing bone morphogenetic protein 4 (BMP4; 50 ng/ml), vascular endothelial growth factor (VEGF; 50 ng/ml) (all Preprotech, USA), 5 μmol/L CHIR99021 (Stemcell Technologies), and 5% protein-free hybridoma medium II (PFHMII) (Thermo Fisher Scientific). Hematopoietic and megakaryocytic differentiation was subsequently induced by supplementation with stem cell factor (SCF) and TPO (50 ng/ml each) and interleukin-3 (IL-3; 25 ng/ml) (all Preprotech), followed by maturation in the presence of SCF and TPO only. The platform produced clinically relevant MK yields under controlled conditions.

Reference

1. Eicke D, Baigger A, Schulze K, Latham SL, Halloin C, Zweigerdt R, *et al.* Large-scale production of megakaryocytes in microcarrier-supported stirred suspension bioreactors. Sci Rep. 2018;8(1):10146. https://doi.org/10.1038/s41598-018-28459-x.

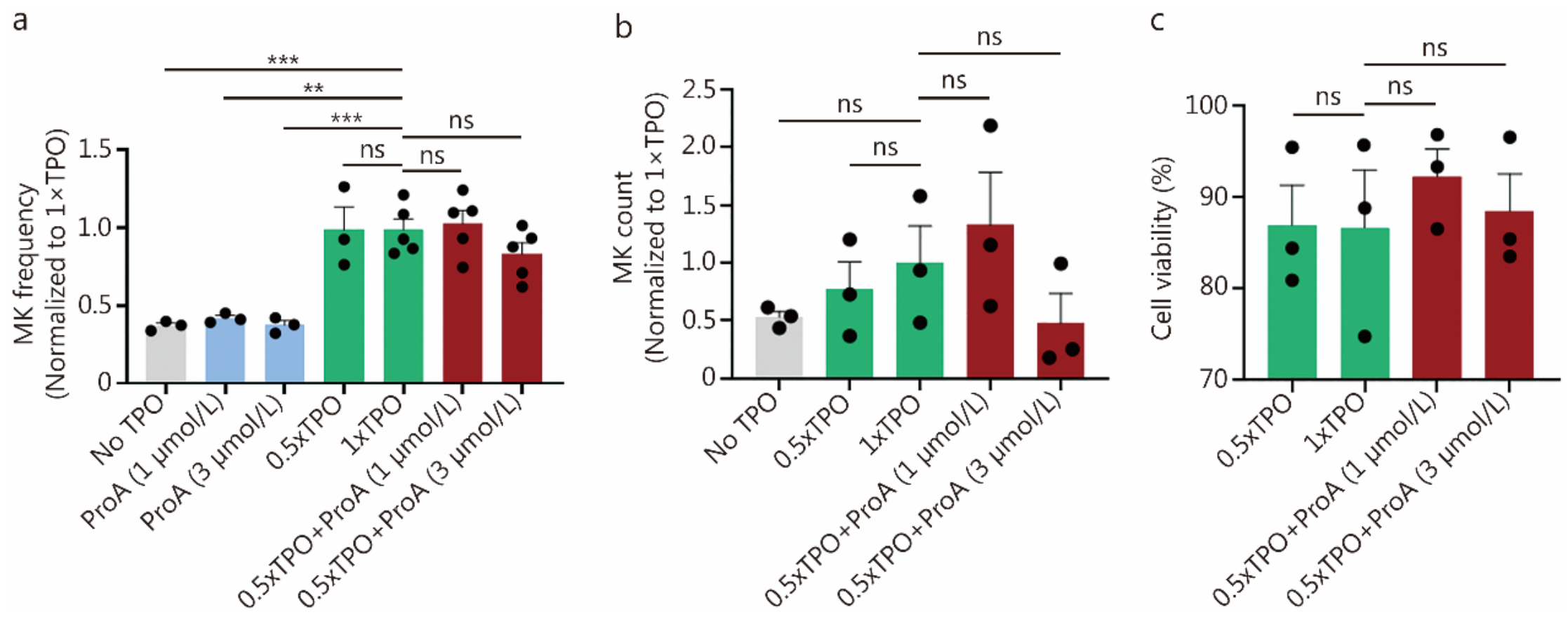

Fig. S1 Evaluation of ProA in iPSC-derived megakaryocyte differentiation under reduced TPO supplementation. **a** Megakaryocyte frequency under standard differentiation conditions (1×TPO) and reduced TPO supplementation [0.5×TPO or No TPO] in combination with 1 or 3 μmol/L ProA. Data are presented relative to the 1×TPO condition. n=3–5. **b** MK count normalized to standard differentiation conditions (1×TPO). n=3. **c** Cell viability in differentiation cultures maintained under standard differentiation conditions (1×TPO), reduced TPO supplementation (0.5×TPO), or 0.5×TPO in combination with 1 μmol/L or 3 μmol/L ProA. n=3. Statistical significance for all data was assessed using an ordinary one-way ANOVA compared to the 1×TPO condition. Data represent mean±standard error of mean (SEM). $^{**}P<0.0021$, $^{***}P<0.0002$, ns non-significant. iPSC. Induced pluripotent stem cell; TPO. Thrombopoietin; ProA. Proanthocyanidin A; MK. Megakaryocyte
